# Host response during *Yersinia pestis* infection of human bronchial epithelial cells involves negative regulation of autophagy and suggests a modulation of survival-related and cellular growth pathways

**DOI:** 10.3389/fmicb.2015.00050

**Published:** 2015-02-13

**Authors:** Farhang Alem, Kuan Yao, Douglas Lane, Valerie Calvert, Emanuel F. Petricoin, Liana Kramer, Martha L. Hale, Sina Bavari, Rekha G. Panchal, Ramin M. Hakami

**Affiliations:** ^1^National Center for Biodefense and Infectious Diseases and School of Systems Biology, George Mason UniversityManassas, VA, USA; ^2^U.S. Army Medical Research Institute of Infectious DiseasesFrederick, MD, USA; ^3^Center for Applied Proteomics and Molecular Medicine, School of Systems Biology, George Mason UniversityManassas, VA, USA

**Keywords:** RPMA, *Yersinia pestis*, host response, signaling pathways, apoptosis and autophagy, phosphorylation changes, cell growth, proteomics

## Abstract

*Yersinia pestis* (Yp) causes the re-emerging disease plague, and is classified by the CDC and NIAID as a highest priority (Category A) pathogen. Currently, there is no approved human vaccine available and advances in early diagnostics and effective therapeutics are urgently needed. A deep understanding of the mechanisms of host response to Yp infection can significantly advance these three areas. We employed the Reverse Phase Protein Microarray (RPMA) technology to reveal the dynamic states of either protein level changes or phosphorylation changes associated with kinase-driven signaling pathways during host cell response to Yp infection. RPMA allowed quantitative profiling of changes in the intracellular communication network of human lung epithelial cells at different times post infection and in response to different treatment conditions, which included infection with the virulent Yp strain CO92, infection with a derivative avirulent strain CO92 (Pgm-, Pst-), treatment with heat inactivated CO92, and treatment with LPS. Responses to a total of 111 validated antibodies were profiled, leading to discovery of 12 novel protein hits. The RPMA analysis also identified several protein hits previously reported in the context of Yp infection. Furthermore, the results validated several proteins previously reported in the context of infection with other Yersinia species or implicated for potential relevance through recombinant protein and cell transfection studies. The RPMA results point to strong modulation of survival/apoptosis and cell growth pathways during early host response and also suggest a model of negative regulation of the autophagy pathway. We find significant cytoplasmic localization of p53 and reduced LC3-I to LC3-II conversion in response to Yp infection, consistent with negative regulation of autophagy. These studies allow for a deeper understanding of the pathogenesis mechanisms and the discovery of innovative approaches for prevention, early diagnosis, and treatment of plague.

## Introduction

The genus Yersinia, a member of the enterobacteriaceae family, consists of 11 species, three of which are pathogenic to humans; *Yersinia pestis (Yp), Yersinia pseudotuberculosis, and Yersinia enterocolitica*. *Y. pestis*, the causative agent of plague, is responsible for a number of major outbreaks throughout history and has caused the deaths of millions of people worldwide. Today, plague is considered a re-emerging disease, with an average of one to two thousand cases of plague and a mortality rate of 8–10% reported to the World Health Organization each year.

For a number of pathogenic bacteria, their ability to alter host protein phosphorylation pathways to allow the subversion of host defenses has been reported (Popova et al., 2010; Younossi et al., 2011; Steiner et al., 2014). In the case of Yp, the pCD1 plasmid encodes for bacterial virulence factors known as Yersinia Outer Proteins (YOPs) that are used to evade the host immune response and manipulate the host cell machinery, as well as proteins essential for the assembly of the Type Three Secretion System (TTSS) (Cornelis, 2002b; Pujol and Bliska, 2005; Plano and Schesser, 2013). The following identified Yop effectors are important in regulating host response, including the modulation of host signaling pathways: YopE, YopH, YopJ, YopK, YopM, YopT, and YpkA (Viboud and Bliska, 2005). Four of them, YopE, H, T, and YpkA have been shown to alter the host cytoskeleton in order to prevent bacterial phagocytosis (Dukuzumuremyi et al., 2000a; Cornelis, 2002b; Sauvonnet et al., 2002; Aepfelbacher et al., 2011; Ke et al., 2013), which has been proposed to be beneficial to Yp because it proliferates much better in the extracellular space (Rosqvist et al., 1991; Cornelis, 2002a; Ke et al., 2013; Plano and Schesser, 2013). Of particular relevance to the impetus for our RPMA study, YopH is a highly potent tyrosine phosphatase and YpkA has serine/threonine kinase activity, and several host targets whose phosphorylation states are modulated by these enzymes have been reported (Black and Bliska, 1997; Dukuzumuremyi et al., 2000b; Navarro et al., 2007; De la Puerta et al., 2009; Rolán et al., 2013). Yop effectors are also involved in altering other host cell signaling pathways, such as the NFκ B and MAPK pathways, as well as modulating host immune responses. For example, YopJ has been shown to interact with upstream targets of the NFκ B pathway to prevent a proinflammatory response and to initiate apoptosis in macrophages (Orth et al., 2000).

The studies of how host pathways are altered during the course of infection with Yp have been limited. While some host targets of specific YOPs have been identified (Black and Bliska, 1997; Mittal et al., 2006; De la Puerta et al., 2009) a comprehensive analysis of how the intracellular communication mechanisms of the host are modulated in the context of whole infection has been lacking. Furthermore, an analysis of how the specific parameters of the infection process such as the multiplicity of infection (MOI), times post infection, or host cell type, may alter the modulation of host pathways has not been yet reported. We previously performed the first quantitative phosphoproteomic analysis of host response to Yp infection that led to the identification of a number of altered host signaling pathways and demonstrated the functional relevance of the AKT pathway during Yp infection of primary human monocytes (Hakami et al., submitted for publication). In this study, we have used the reverse phase protein microarray (RPMA) technology to perform a comparative quantitative analysis of the modulation of host phosphorylation under a variety of different infection parameters, including different times post infection (p.i.), different cell types, and infection of host cells with both a fully virulent and a derivative avirulent Yp strain. In the RPMA methodology, the analytes are robotically spotted onto nitrocellulose coated glass slides as capture molecules (hence the phrase “reverse phase”) and, after blocking, the slides are probed with specific primary antibodies that have been validated for single-band specificity by Western immunoblotting and for use with RPMA. Primary antibody binding is detected using appropriate biotinylated secondary antibody followed by streptavidin-conjugated fluorophore treatment for detection. Each microarray consists of a self-contained assay comprised of triplicate samples, controls and calibrators that are analyzed with one class of antibody and amplification chemistry. RPMA is a novel and validated technology that has been gaining recognition for its ability to study complex cellular signaling networks through quantitative analysis of protein levels and post translational modifications. It has been widely used in cancer research, leading to discovery of changes in cellular signaling pathways in comparative studies of normal vs. cancer cells (Wulfkuhle et al., 2012), and more recently has also been applied for the studies of a variety of other diseases, such as neurodegenerative diseases, cardiovascular disease, and infectious diseases. The RPMA technology affords the use of a quantitative and high throughput platform for simultaneous analysis of multiple signaling pathways within the cell in order to provide an overall picture of the signaling architecture of the cell for any given state, abilities that are not practically afforded by the more traditional platforms such as DOT blots or western analysis (Mueller et al., 2010).

Since an aerosol route of infection is of most concern within the context of a biological attack, we used human lung epithelial cells as one of our infection models and present the results of our study with this cell type. In addition to validation of published reports on host signaling in response to Yp, we report here the identification of a number of novel proteins that show significant change in either phosphorylation levels or total protein levels and that have not been previously reported within the context of Yp infection. Furthermore, since several previously reported host response findings that we have validated here had been performed in the context of recombinant protein expression and transfections, our results provide evidence that these host proteins are indeed involved in the context of whole infection. We also provide a comparative profiling analysis between changes observed during infection with the fully virulent Yp strain and a derivative avirulent Yp strain and also changes that are observed in response to heat-killed bacteria or LPS treatment and are therefore independent of TTSS. Finally, based on our RPMA analysis of the human lung epithelial cells, and also analyses of the localization of p53 protein and LC3-I to LC3-II conversion during infection with Yp, we propose and discuss a model of negative regulation of the autophagy pathway as part of the host response to Yp infection in human lung epithelial cells.

## Materials and methods

### Bacterial strains and reagents

Bacterial strains used in this study were virulent *Yersinia pestis* CO92 and a derivative avirulent strain, *Yersinia pestis* CO92 (Pgm-, Pst-) (a gift from Drs. Susan Welkos and Christopher Cote, USAMRIID), that is pigmentation (pgm)-deficient and cured of the plasminogen-activator-encoding pPst plasmid (Welkos et al., 2002; Jenkins et al., 2009; Kota et al., 2013). Treatment with the heat-killed version of *Y. pestis* CO92 strain (heat-killed at 65°C for 30 min) was also performed. For infections, bacterial strains were streaked onto Sheep Blood Agar (SBA) plates from a frozen stock and grown at 28°C. A single colony was isolated and used to inoculate cation-adjusted Mueller-Hinton broth (CAMHB) and grown overnight at 28°C to use for infecting cells. Overnight cultures were enumerated using OD_600_ readings (an OD_600_ reading of 1 is equivalent to 5.8 × 10^8^ CFU). Antibodies used for the RPMA analysis are listed in Supplementary Table 1, along with dilution factors used and vendor information. All the antibodies had been previously validated for RPMA use. For each Western blot validation, and also the LC3 Western analysis, the identical antibody used for RPMA was utilized.

### 16HBE14o- cell infections

Immortalized human airway epithelial cells (16HBE14o-) were purchased from Dr. D.C. Greunert (California Pacific Medical Center Research Institute, San Francisco, CA).16HBE14o- (HBE) cells were grown in Bronchial Epithelial Cell Basal Medium (Clonetics™ BEGM™ BulletKit™ (CC-3170) supplemented with: BPE, 2 ml; Hydrocortisone, 0.5 ml; hEGF, 0.5 ml; Epinephrine, 0.5 ml; Transferrin, 0.5 ml; Insulin, 0.5 ml; Retinoic Acid, 0.5 ml; Triiodothyronine, 0.5 ml (Lonza, Walkersville, MD). Cells were cultured in 6 well plates and 2 × 10^6^ cells per well were infected at a MOI of 10 with either fully virulent strain of *Y. pestis*, CO92, or a derivative avirulent *Y. pestis* strain CO92 (Pgm-, Pst-), or were treated with heat-killed *Y. pestis* CO92. Untreated, and *E. coli* lipopolysaccharide (LPS)-treated cells (100 ng/ml), were also included as controls. Cells were harvested at 30 min, 1, 4, and 8 h post infection, washed with 1 × PBS and then lysed using lysis buffer: 30 ml 2× Novex Tris-Glycine SDS Sample Loading Buffer (Invitrogen), 20 ml T-PER Tissue Protein Extraction Reagent (Thermo Scientific), 200 μl 0.5 M EDTA pH 8.0, 1X Complete Protease Inhibitor Cocktail (Roche), 80 μl 0.1 M Na_3_VO_4_, 400 μl 0.1 M NaF, 1.3 ml 1 M DTT. Samples were then stored at −20°C.

### Bacterial uptake and intracellular growth measurements

1 × 10^5^ 16HBE14o-cells were infected with CO92 (Pgm-, Pst-) strain at MOI of 10 and incubated at 37°C for 2 h. The cells were subsequently incubated with 50 μg/ml gentamycin for 1 h at 37°C to eliminate the extracellular bacteria. The cells were then washed and resuspended in BEGM media containing 8 μg/ml Gentamycin. At designated time points post infection (0, 8, and 24 h p.i.) cells were washed and then lysed by incubating in 0.2% Triton-× 100 for 5 min at 37°C. Dilutions of the cell lysates were spread onto SBA plates and incubated at 37°C to allow bacterial colony growth. Bacterial colonies were enumerated and CFU calculations were used to measure the levels of bacterial uptake and intracellular growth.

### RPMA analysis

Sample protein levels were first determined using pre-printed slides containing all the samples as well as a BSA curve and stained with Sypro to calculate total protein levels in the samples. Equivalent total protein amounts for samples were arrayed onto nitrocellulose coated glass slides by direct contact printing using a high resolution 2470 arrayer (Aushon Biosystems, Brillerica, MA). RPMA analysis was performed as previously described (Federici et al., 2013). Samples were printed in triplicate and averages were calculated for analysis. Sample slides were probed with 111 different antibodies against either total or phosphorylated forms of proteins that are involved in various cell signaling pathways. A complete list of all the antibodies used for this analysis and categorized by functional relevance is provided in Supplementary Table 1.

### RPMA data analysis

For each specific post-infection time point, the RPMA results for the different infection or treatment conditions were normalized to the control group (uninfected and untreated) in order to calculate relative fold-change levels. For total protein levels, a 2-fold increase/decrease was set to be significant and for phosphorylation levels the significant fold increase/decrease was set to 1.5. Proteins that showed significant fold change were then separated into three main categories based on the respective infection or treatment condition that elicited the observed changes (Tables 1, 2). Extensive literature searches were performed using Pubmed for all the proteins that showed significant fold change, in order to identify novel proteins that have not been previously reported to play a role during *Y. pestis*-host interactions (Table 3).

**Table 1:**
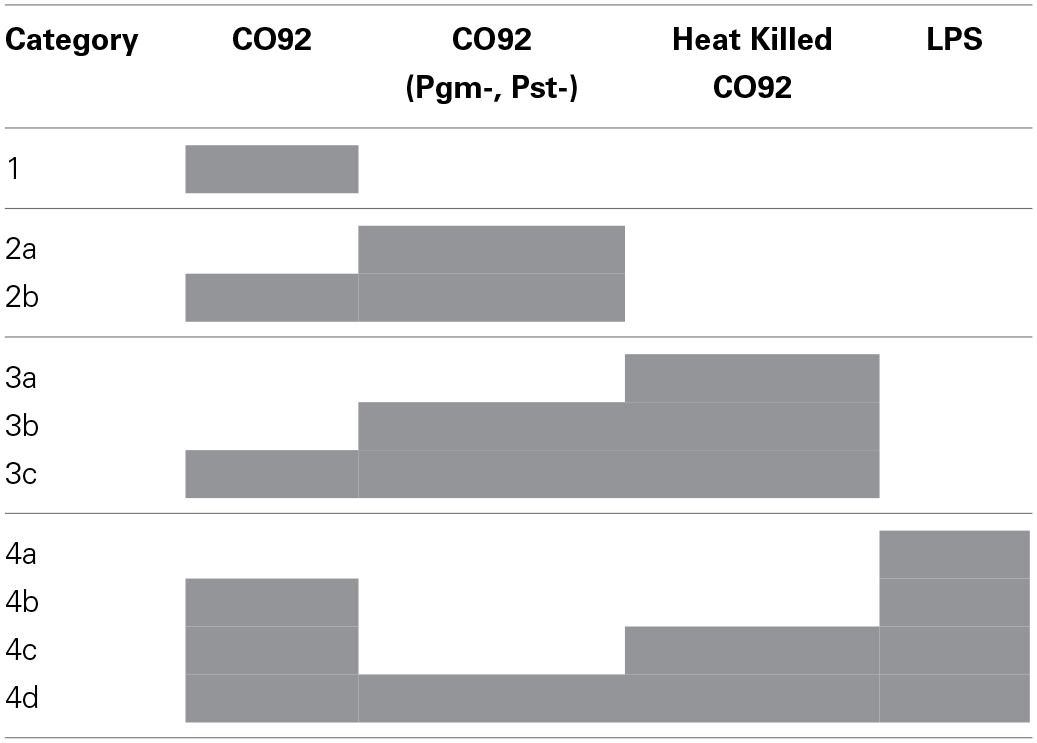
**Categorizations of the RPMA protein hits**.

*Proteins that showed a relative fold change on RPMA arrays of 1.5 and higher for total protein levels, or 2-fold and higher for phosphorylation levels, were divided into 4 main categories based on treatment conditions, as indicated by gray colored boxes. Each category was subdivided to account for the observed changes that occurred in response to more than one treatment condition. Category (1) designates protein hits that were identified only in response to infection with the virulent Y. pestis CO92 strain. Category 2 proteins designate significant responses to either the avirulent Y. pestis CO92 (Pgm-, Pst-) strain alone (2a), or to both the avirulent and virulent strains (2b). Category 3 proteins designate significant responses to either heat-killed Yp (HK-CO92) only (3a), or to both HK-CO92 and the avirulent strain (3b), or to all three HK-CO92, avirulent strain, and virulent strain treatments (3c). Category 4 proteins designate significant responses to LPS alone (4a), or to both LPS and one or more of the other treatment conditions, which includes LPS and Y. pestis CO92 (4b), LPS, Y. pestis CO92, and HK-CO92 (4c), or all 4 treatments (4d)*.

**Table 2:**
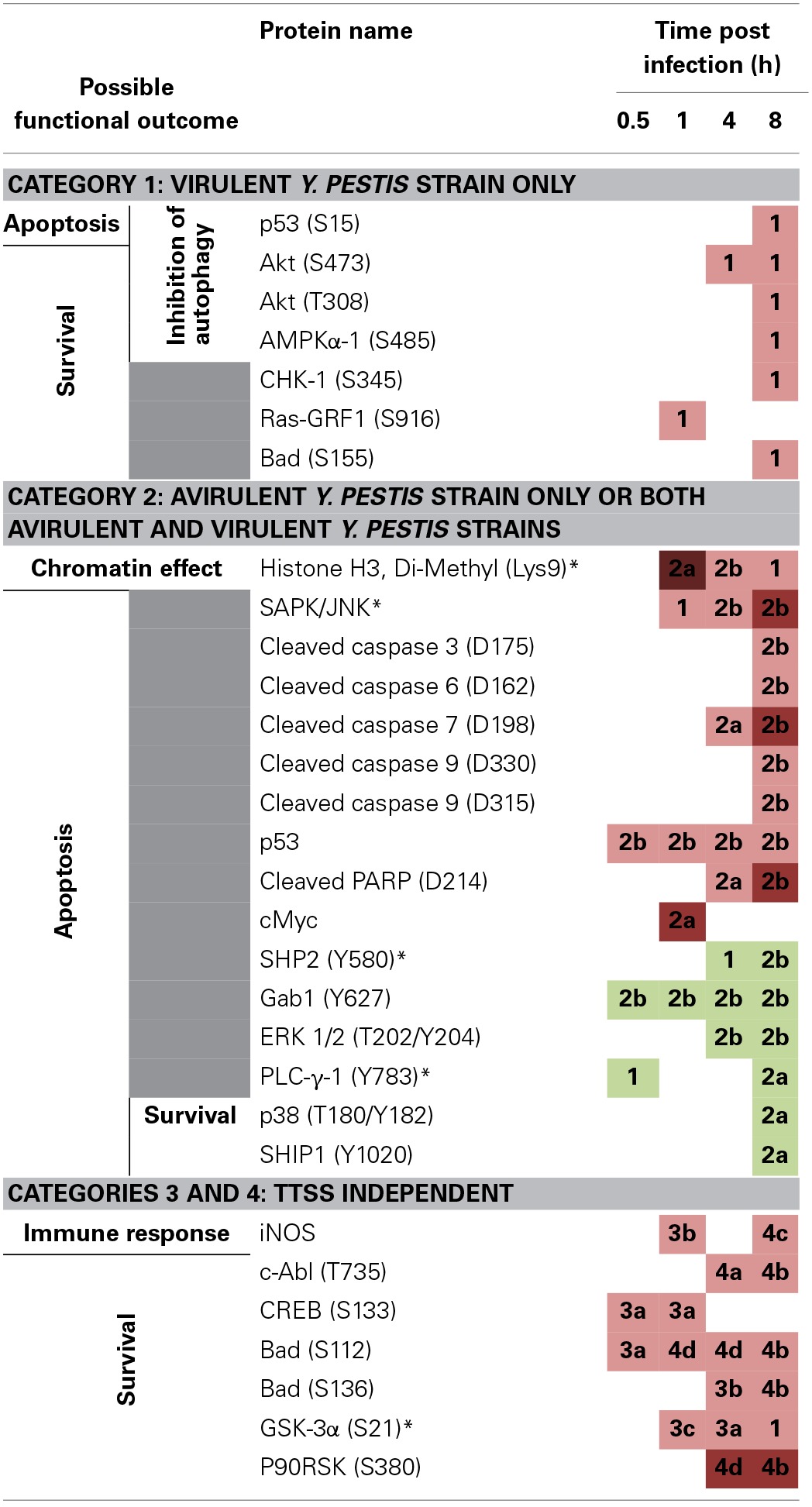
**Protein hits identified by RPMA and their proposed functions in the context of Yp infection**.

Proteins with significant changes relative to the control condition are listed. For each protein, the observed changes at specific post infection times are indicated. Green color represents significant decrease (≥2-fold decrease for total protein levels and ≥1.5-fold decrease for phosphorylation events) and red color represents significant increase. Light red represents a 1.5 to 3-fold increase for phosphorylation events and 2 to 3-fold increase for total protein levels. Medium red represent a 3 to 6-fold increase for either phosphorylation or total protein level changes. Dark red color, assigned only to one protein at the 1 h time point [Histone H3, Di-Methyl (Lys 9)], represents an 11-fold increase in total level of this protein. Category designations for each time point has been indicated based on the categorization scheme of Table 1. The left side columns indicate possible functional outcomes of the observed protein changes based on the known roles of the proteins. Asterisks denote proteins that fall into different categories at different time points.

**Table 3:** **Discovery of novel hits and validation of previous findings in the context of Yp infection**.

**Novel proteins**	**Functional relevance**	**Category**
Bad (S112)	Apoptosis/**Survival**	3, 4
Bad (S136)	Apoptosis/**Survival**	3, 4
Bad (S155)	Apoptosis/**Survival**	1
Cl-Caspase-6 (D162)	**Apoptosis**/Survival	2
cMyc	**Apoptosis**/Survival, Growth	2
c-Abl (T735)	**Apoptosis**/Survival, Growth	4
p53	**Apoptosis**/Survival, **Autophagy Regulation**, Cell cycle	2
p53 (S15)	**Apoptosis**/Survival, **Autophagy Regulation**, Cell cycle	1
AMPK-α1 (S485)	Apoptosis/**Survival**, **Autophagy Regulation**, Homeostasis	1
SHIP1 (Y1020)	Apoptosis/Survival, Growth	2
SHP2 (Y580)	**Apoptosis**/Survival, Growth	1, 2
Chk-1 (S345)	Apoptosis/**Survival**, Growth, Cell cycle	1
iNOS	Immune response	3, 4
Histone H3, Di-methyl (Lys9)	Chromatin structure	1, 2
Ras-GRF1 (S916)	Cytoskeleton modulation	1
**Reported proteins**	**Functional relevance**	**Reference(s)**
**REPORTED FOR *YERSINIA PESTIS***		
Cl-Caspase-3 (D175)	Caspase activity low with Yp Kim YopJ	Zheng et al., 2012
	Pla degrades FasL, which decreases Caspase-3 activation	Caulfield et al., 2014
	YopK contributes to Caspase-3 cleavage	Peters et al., 2013
Cl-Caspase-7 (D198)	Caspase-7 activity low with Yp Kim YopJ	Zheng et al., 2012
	Pla degrades FasL, which decreases Caspase-7 activation	Caulfield et al., 2014
Cl-PARP (D214)	Cleaved-PARP indicates caspase pathway activation by Yp	Zheng et al., 2012
**REPORTED FOR OTHER YERSINIA SPECIES**		
Akt (S473), Akt (S308)	*Y. enterocolitica* YopH inactivates Akt pathway	Sauvonnet et al., 2002
Cl-Caspase-9 (D330) Cl-Caspase-9 (D315)	*Y. enterocolitica* YopP activates Caspase-9 through Bid	Denecker et al., 2001
CREB (S133)	YopJ of *Y. pseudotuberculosis* can block CREB activation	Meijer et al., 2000
GSK-3α (S21)	*Y. enterocolitica* YopH modulates PI3K/Akt/GSK-3α pathway	Sauvonnet et al., 2002
P90RSK (S380)	*Y. enterocolitica* YopM induces sustained RSK activation	Hentschke et al., 2010
**REPORTED USING RECOMBINANT PROTEINS OR TRANSFECTED CELLS**		
Akt (S473), Akt(S308)	Akt is involved in *Y. pseudotuberculosis* entry into host cells	Uliczka et al., 2009
ERK 1/2 (T202/Y204)	rF1 induces phosphorylation of ERK1/2 in macrophages	Sharma et al., 2005b
	rYopB and rLcrV inhibit expression of phospho-ERK 1/2	Sodhi et al., 2004
PLC-γ-1 (Y783)	PLC-γ-1 plays a role in *Y. pseudotuberculosis* cell entry	Uliczka et al., 2009
Gab1 (Y627)	Transfected YopH of Yp associates with Gab1	De la Puerta et al., 2009
SAPK/JNK	JNK plays a role in rF1 induced activation of macrophages	Sharma et al., 2005a

*Novel protein hits discovered through the RPMA analysis and protein hits that have been previously implicated for relevance to the process of Yersinia infections are listed. For the novel hits, the affected pathways in which they are known to be involved are indicated; the bold type highlights those pathways that are implicated by the specific RPMA changes observed for these proteins during Yp infection. Previously reported proteins are separated based on whether the studies had been performed in the context of Yp infection or infection with other Yersinia species, as well as studies that were based on treatment with recombinant Yp proteins or cell transfections*.

### Western blot analysis

RPMA findings were validated by Western blot analysis for 6 selected proteins that displayed significant fold change on RPMA arrays, including cleaved PARP (D214) and p53 (S15). 15 ul of cell lysate in lysis buffer was boiled for 10 min at 96°C and loaded onto a 4–20% bis-tris polyacrylamide gel (Invitrogen). The gel was run at 200 V for 40 min, transferred to nitrocellulose membrane using the iBlot Gel Transfer Device (Invitrogen), and blocked overnight in 5% dry milk in PBS-T. The same primary antibodies used for the RPMA analysis (Supplementary Table 1) were added at appropriate dilutions in 5% dry milk/PBS-T for 1 h at room temperature, followed by a 1 h incubation with an appropriate secondary antibody (goat-anti Rabbit or goat-anti Mouse) in PBS-T. The protein bands were visualized using SuperSignal West-Femto Maximum Sensitivity Substrate (Pierce). The blots were imaged using Chemidoc XRS System (BioRad, CA). Actin controls were used to normalize total protein levels for different samples relative to one another.

LC3-B Western blots were performed as follows: 20 μl of total cell extract from uninfected/untreated cells, the cells infected with Yp CO92, and LPS-treated cells were run on a 4–12% bis-tris polyacrylamide gel (Invitrogen) at 200 V for 40 min, and transferred to nitrocellulose membrane using the iBlot Gel Transfer Device (Invitrogen). The membrane was blocked overnight in 5% dry milk in PBS-T, and then washed 3 times with PBS-T and incubated with the LC3B primary antibody (1:1000) for 1 h at room temperature. Following 3 more washes with PBS-T, the secondary antibody was added at 1:5000 dilution and the blot was incubated at room temperature for 1 h. After three washes with PBS-T to remove excess secondary antibody, the protein bands were visualized using SuperSignal West-Femto Maximum Sensitivity Substrate (Pierce). The blots were imaged using Chemidoc XRS System (BioRad, CA). Actin controls were used to normalize total protein levels for different samples relative to one another.

### p53 (S15) immunofluorescence staining

Immunofluorescence staining was performed to analyze the cellular localization of activated p53 protein (phosphorylated at residue S15). Eight well chamber slides (EW-01838; Thermo Scientific) were seeded with HeLa cells at a cell density of 10,000 cells per well. The following day, the cells were either left untreated and uninfected (control), or were infected with *Y. pestis* CO92 (Pgm-, Pst-) at MOI of 10 for 8 h to match the exact RPMA conditions that showed an effect, or were treated with Doxorubicin as described previously (Kurz et al., 2004). Cells were then fixed with 1% paraformaldehyde for 15 min at 37°C, and then rehydrated with PBS for 5 min at room temperature. The cells were subsequently permeabilized using 0.1% Triton X-100 (Sigma) for 10 min at room temperature and blocked with 2% BSA (Sigma) for 1 h at room temperature. The cells were then washed with PBS three times and incubated with the same anti-p53 (S15) primary antibody used in the RPMA study (Cell signaling; catalog number 9282) at 1:500 dilution for 1 h at room temperature. Subsequent PBS washes were performed, followed by incubation with Alexa-Fluor 488 Goat-anti Mouse secondary antibody at 1:1000 dilution for 1 h at room temperature. The cells were then washed three times with PBS and slides were mounted using Vectashield with DAPI (Vector Laboratories: Burlingame, Ca). Control conditions were also included side by side to account for any background signal; for this, cells received an identical treatment except that addition of either the primary antibody or the secondary antibody was omitted and an equivalent volume of PBS was added instead. The slides were imaged using a Nikon Eclipse TE2000-U confocal microscope.

## Results

### RPMA analysis of HBE cells infected with *Yersinia pestis*

In order to study host cell signaling pathways that are altered during Yp infection, including pathways that are modulated independently of the TTSS mechanism, we compared host response of 16HBE14o- cells (human bronchial epithelial cells) infected with the fully virulent strain CO92 with the response to infection with a derivative avirulent strain, CO92 (Pgm-, Pst-). Host response to treatment with heat-inactivated CO92 or LPS was also analyzed side by side. Untreated and uninfected cells were also included as controls. For the avirulent strain CO92 (Pgm-, Pst-), we found that about 5% of the bacteria were taken up by the 16HBE14o- cells during infection at MOI of 10. While a 4-fold increase in the number of intracellular bacteria was observed by 8 h post infection, the bacteria stopped growing after this time and the same number of intracellular bacteria as the 8 h time point was observed at 24 h post infection. For our RPMA analysis, 30 min, 1, 4, and 8 h post infection times were analyzed using 111 different host-specific pre-validated antibodies, in order to quantify changes in protein phosphorylation levels or total protein levels (Table S1). The antibodies were selected to allow a wide range functional analysis of host cellular pathways (Table S1), and also included those that are known or suggested from previous studies to be affected during Yp infection, such as apoptosis, cytoskeletal modulation, inflammation, immune response, and autophagy.

**Figure 1:**
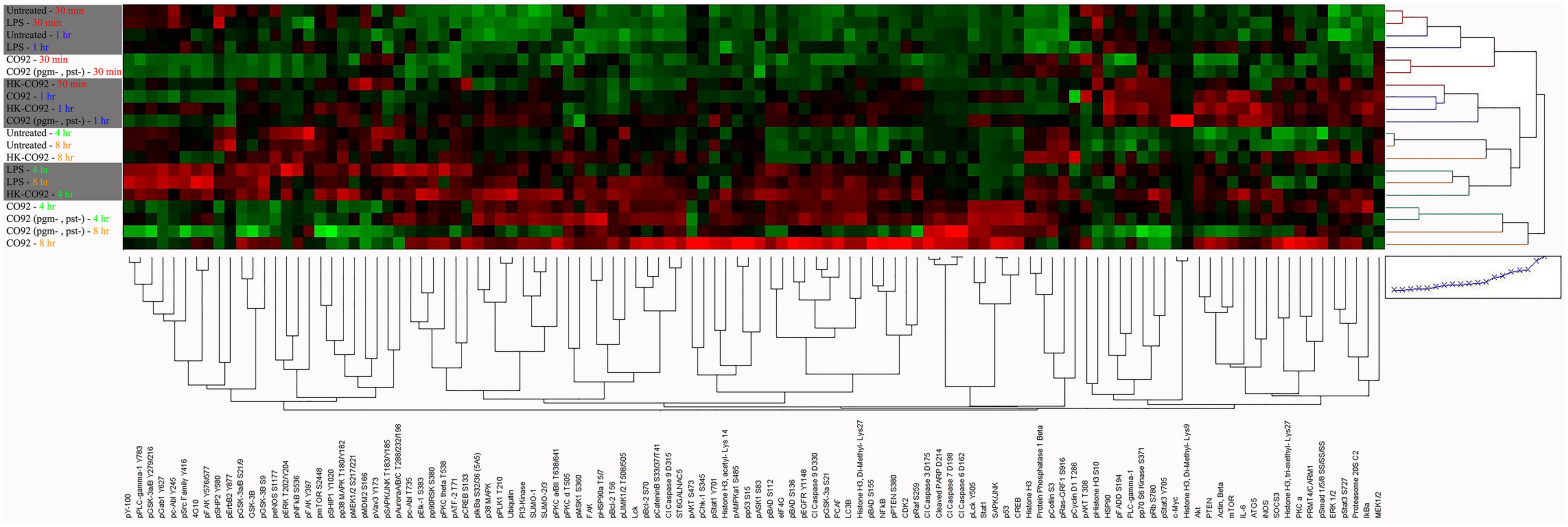
**Heat map of RPMA results**. The Heat map shows unsupervised hierarchical 2-way clustering analysis of all the samples analyzed by RPMA. Rows represent the different samples used in the RPMA analysis and the columns represent the different antibodies that were tested. Relative signal intensities were assigned based on comparison with the lowest signals on the arrays. Red color depicts higher signal values and green color depicts lower signal values; color intensity indicates the strength of signal.

The heat map in Figure 1 presents an unsupervised hierarchical 2-way clustering analysis of the RPMA data. According to this analysis, similar host response profiles were observed between the virulent and avirulent Yp strains, particularly at the later time points (4 and 8 h post infection). On the other hand, samples treated with heat-killed bacteria group with the untreated and LPS-treated controls group. While the host response profile to live bacteria appears distinct from that of the control samples as a whole, there is no discernable clustering of the proteins according to functional categories or pathways with regard to treatment with live bacteria versus the control samples.

### Identification and categorization of proteins with significant fold change

To identify changes in the protein expression levels or specific protein phosphorylation during Yp infection, relative fold changes were calculated by normalizing the samples with respect to the uninfected and untreated control samples for each time point. The thresholds for significant fold change was set to 1.5-fold or higher for phosphorylation events and 2-fold or higher for total protein levels. The identified hits were then categorized into 4 distinct categories that allow distinguishing the changes that are unique to infection with the fully virulent strain (CO92) from those that occur in response to infection with the avirulent strain, or significant changes that are observed during LPS and heat-killed bacterial treatments (Table 1). This categorization also highlights significant changes that are not unique to any particular treatment and are observed during two or more of the treatment conditions that we have tested.

Category 1 proteins are those that showed a significant change only in response to fully virulent Yp CO92 strain. Categories 2a and 2b are those that showed a significant change in response to either the attenuated strain alone or in response to both the attenuated and virulent strains, respectively. Category 3 proteins designate those that showed a significant change in response to the heat-inactivated CO92, and are subdivided into subcategories 3a, 3b, and 3c. Category 3a refers to significant changes in response to the heat inactivated treatment alone; categories 3b refers to significant changes in response to both the heat inactivated bacteria and the avirulent strain, and category 3c designates significant responses that are observed for heat inactivated bacteria, the avirulent strain, and the virulent strain. Category 4 proteins signify those that showed significant fold changes in response to LPS treatment, and are subdivided into subcategories 4a, 4b, 4c, and 4d. Category 4a refers to significant changes in response to LPS treatment only. Category 4b refers to significant changes in response to both the LPS treatment and infection with the virulent strain. Category 4c designates those proteins that showed significant fold change in response to LPS treatment, treatment with the heat-inactivated bacteria, and infection with the virulent strain. Category 4d designates proteins that showed significant change in response to all four treatments conditions. Of all the proteins analyzed by RPMA, 25 proteins were identified to exhibit significant fold changes, as defined by the assigned threshold values for significance (Table 2).

Category 1 consists of 6 proteins that show significant phosphorylation increases (AKT-S473 and-T308, AMPKa1-S485, CHK1-S345, Ras-GRF1-S916, p53-S15, and BAD-S155). These proteins are known to be involved in multiple intracellular signaling pathways, and based on their established roles the consequences of the observed RPMA changes suggest apoptotic/survival and/or autophagy-related functional outcomes (Table 2). Therefore, the RPMA results show that during the early phases of Yp infection the apoptosis/survival pathways and mechanisms involved in negative regulation of autophagy are among the very key signaling mechanisms that become engaged. Within category 1, with the exception of Ras-GRF1(S916) that was altered within the first hour post infection, the remainder of the proteins showed significant changes later, at 8 h post infection.

Category 2 contains 15 proteins, 9 of which show significant increases in either protein levels or phosphorylation post infection (Histone H3 Di-methyl (Lys9), SAPK/JNK, Cleaved caspase 3-D175, Cleaved caspase 6-D162, Cleaved caspase 7-D198, Cleaved caspase 9-D330 and -D315, p53, Cleaved PARP-D214, and c-Myc) and 6 of which show a significant decrease in phosphorylation (SHP2-Y580, Gab1-Y627, ERK 1/2-T202/Y204, PLC-γ-1-Y783, p38-T180/Y182, and SHIP1-Y1020). Except for Histone H3 Di-methyl (Lys9), all the other proteins in this category are known to be involved in the apoptosis/survival pathways and the specific phosphorylation changes shown by RPMA for 12 of these proteins point to an overall induction of apoptosis following Yp infection (Table 2). This striking observation suggests that within category 2 (which designates changes elicited by the avirulent Yp strain alone, or by both the avirulent and the virulent strains) a major signaling response of the host is the activation of cell death pathways.

The remaining 6 host proteins from the total of 25 identified hits belong to category 3 (treatment with heat-killed Yp) and/or category 4 (LPS treatment). Because these treatment conditions are devoid of TTSS function, the host protein changes observed in response to these treatments cannot be caused by TTSS-dependent mechanisms, but clearly extensive future studies are required to identify the mechanisms that cause the observed host changes during these treatment conditions. The live Yp strains also elicited significant changes for some of these proteins (listed for subcategories 3b, 3c, and 4b, 4c, and 4d). The 6 proteins, iNOS, cAbl-T735, CREB-S133, BAD-S112 and -S136, GSK-3α-S21, and P90RSK-S80, all show significant fold increase, with some as early as 30 min post infection. For category 3, the increased phosphorylations of the BAD protein at S112 and S136 residues lead to an inhibition of its apoptotic functions and therefore are predicted to promote cell survival. Furthermore, the observed significant increases in iNOS levels, activation of CREB by phosphorylation at S133, and inhibition of GSK-3α by phosphorylation at S21, are all predicted to lead to survival outcomes based on the known functions of these proteins (Park et al., 2005; Song et al., 2005; Kotliarova et al., 2008). Therefore, our RPMA results point to a coordinated early induction of survival during Yp infection of lung epithelial cells that can occur through TTSS-independent mechanisms.

### Validation of RPMA results by Western blots

**Figure 2:**
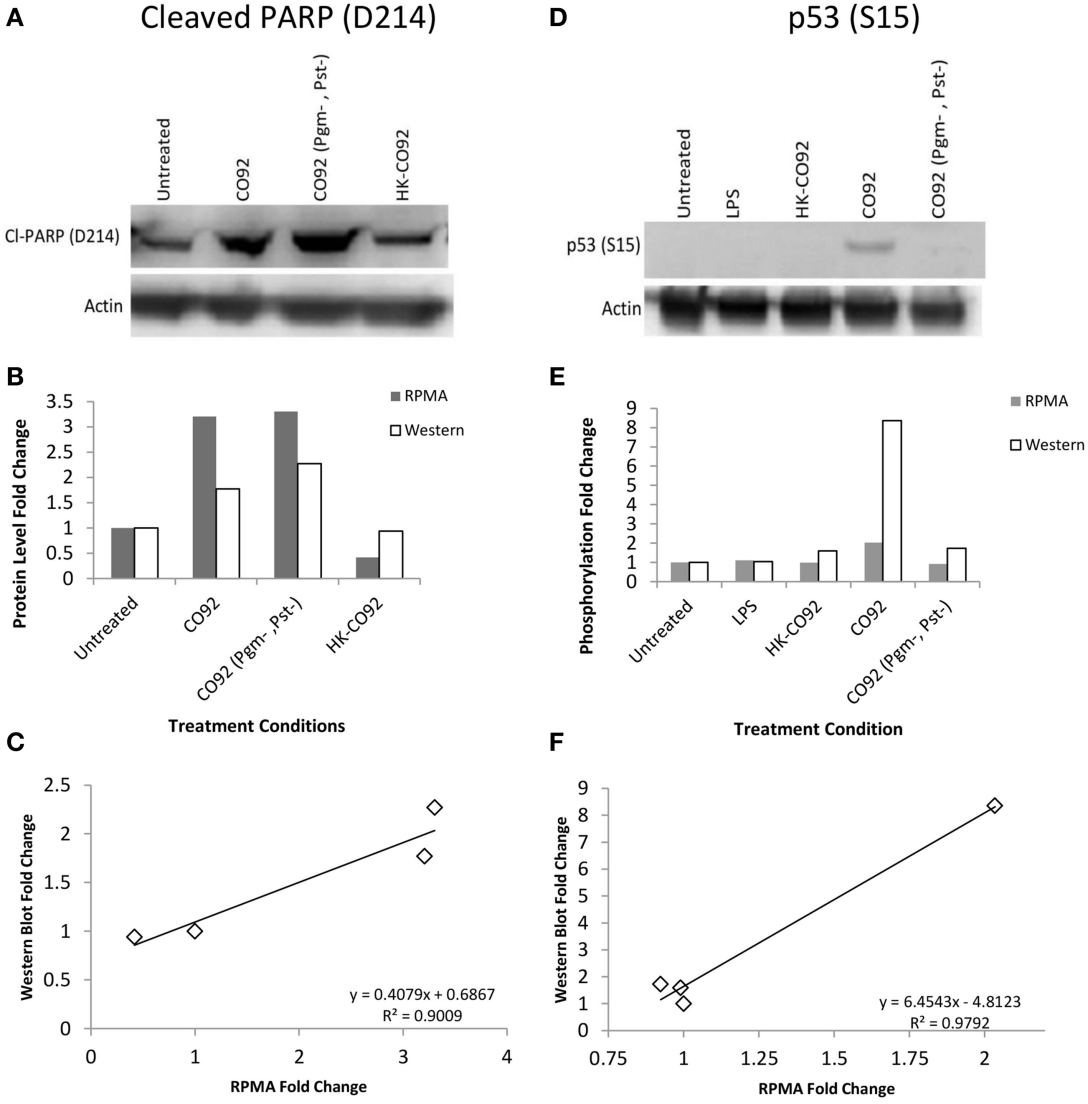
**Western blot validation of RPMA results. (A,D)** Western blot analysis of human bronchial epithelial (HBE) cells for control condition (uninfected and untreated), treatment with heat-killed Yp CO92 (HK-CO92) or with LPS, and infection with Yp CO92 or Yp CO92 (Pgm-, Pst-). The blot in **(A)** was probed with the same antibody that was used for the RPMA analysis of cleaved PARP (D214), and the blot in **(D)** was probed with the same antibody against p53 (S15) that was used for RPMA analysis. Actin levels were also probed for all samples to serve as loading control and allow normalization of the signals for measuring fold changes relative to the control condition. **(B,E)** Bar graphs showing protein level fold changes of cleaved-PARP (D214) in **(B)**, and phosphorylation fold changes of p53 (S15) in **(E)**, for both the RPMA and Western blot analyses of the treatment conditions. For comparison purposes, the value for the control condition (uninfected and untreated) was set at 1. **(C,F)** Two-way scatter plot showing positive correlation between the RPMA results and the Western blot results for Cleaved-PARP (D214) and p53 (S15).

Even though RPMA antibodies have already been pre-validated and reported in previous RPMA publications (Popova et al., 2010; Wilson et al., 2010; Younossi et al., 2011; Sereni et al., 2013), we nevertheless performed another tier of validation of our RPMA results by Western blot analysis of selected hits, testing both the same sample set that was used for the RPMA analysis and a biological replicate sample set. Multiple RPMA hits were validated by Western blot and two representative examples are presented in Figure 2, for cleaved PARP (D214) and p53 (S15) proteins. Our Western analysis confirms the RPMA findings of an increase in PARP (D214) levels and activation of p53 by increased phosphorylation at S15 residue during Yp infection (Figures 2A,D). For different treatment conditions, fold changes in protein levels or phosphorylation levels were measured by comparing the band intensities to untreated controls, and the comparisons between the RPMA and Western fold changes for Cleaved PARP (D214) and p53 (S15) are presented (Figures 2B,E). Two-way scatter plot analysis shows strong positive correlation between the western blot and RPMA results, with a R^2^ value of 0.9 for cleaved PARP (D214) and a R^2^ value of 0.98 for p53 (S15) (Figures 2C,F).

### Discovery of novel proteins and confirmation of previous findings within the context of *Y. pestis* infection

Our RPMA analysis of the human lung epithelial cells yielded 25 proteins that displayed significant changes in either site-specific phosphorylation or total protein levels in response to Yp infections; a complete list of these proteins as well as the pathways engaged by the novel hits are shown in Table 3. After a comprehensive literature search, 12 of the 25 hits were identified to be novel findings, and have not been previously reported for Yp infection. Our study also led to the identification of several hits that have been previously reported, although the majority of them have been reported either in the context of infection with other Yersinia species or within the context of using recombinant proteins and transfections with specific Yp components (Table 3). Thus, our identification of several previously reported changes not only provides an important level of validation for our RPMA findings but also places a number of the previous reports within the more relevant context of whole infection with Yp, which we have performed using a relevant model cell type and under both fully virulent and avirulent infection conditions.

Two of the main pathways that are highlighted by our novel hits are the autophagy and survival-related pathways. In addition, cell cycle and growth regulation, and regulation of chromatin structure, are implicated. The identification of these novel hits by RPMA helps to fill in the current gap of knowledge with regard to the overall signaling network changes that occur during early response to infection with Yp. For instance, as discussed in the next section, our discovery of specific changes in several proteins that regulate autophagy, together with initial mechanistic results, leads to a model of negative regulation of autophagy during early response of human lung epithelial cells to Yp infection.

### Evidence for negative regulation of autophagy

**Figure 3:**
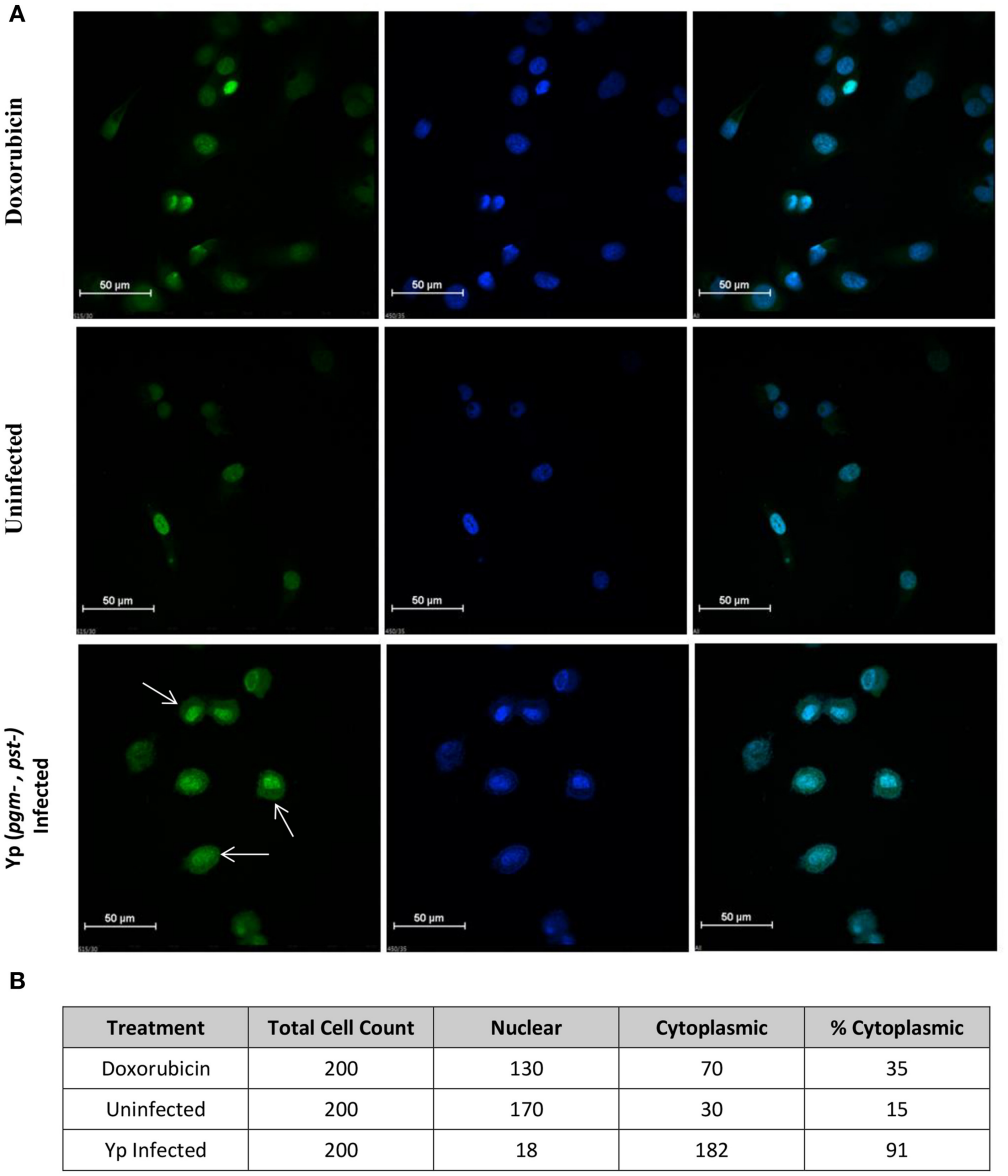
**p53 localization in the cytoplasm of Yp-infected cells. (A)** Confocal microscopy image of HeLa cells for analysis of p53 localization. Top panel shows cells treated with Doxorubicin at 1 μM for 1 h at 37°C to induce p53 expression and serve as positive control. Middle panel shows uninfected cells and bottom panel shows cells infected with Yp CO92 (Pgm-, Pst-) for 8 h. Green fluorescence (left column) indicates activated p53 signal, obtained by probing with p53 (S150) antibody, and blue signal (middle column) corresponds to DAPI nuclear stain. The right column shows superimpositions of the two signals. White arrows show p53 localization in the cytoplasm of the infected cells compared to uninfected and Doxorubicin-treated cells. **(B)** For each treatment condition, cells were scored for cytoplasmic or nuclear localization of p53 (S15) and percentages of cells showing cytoplasmic signal were calculated.

**Figure 4:**
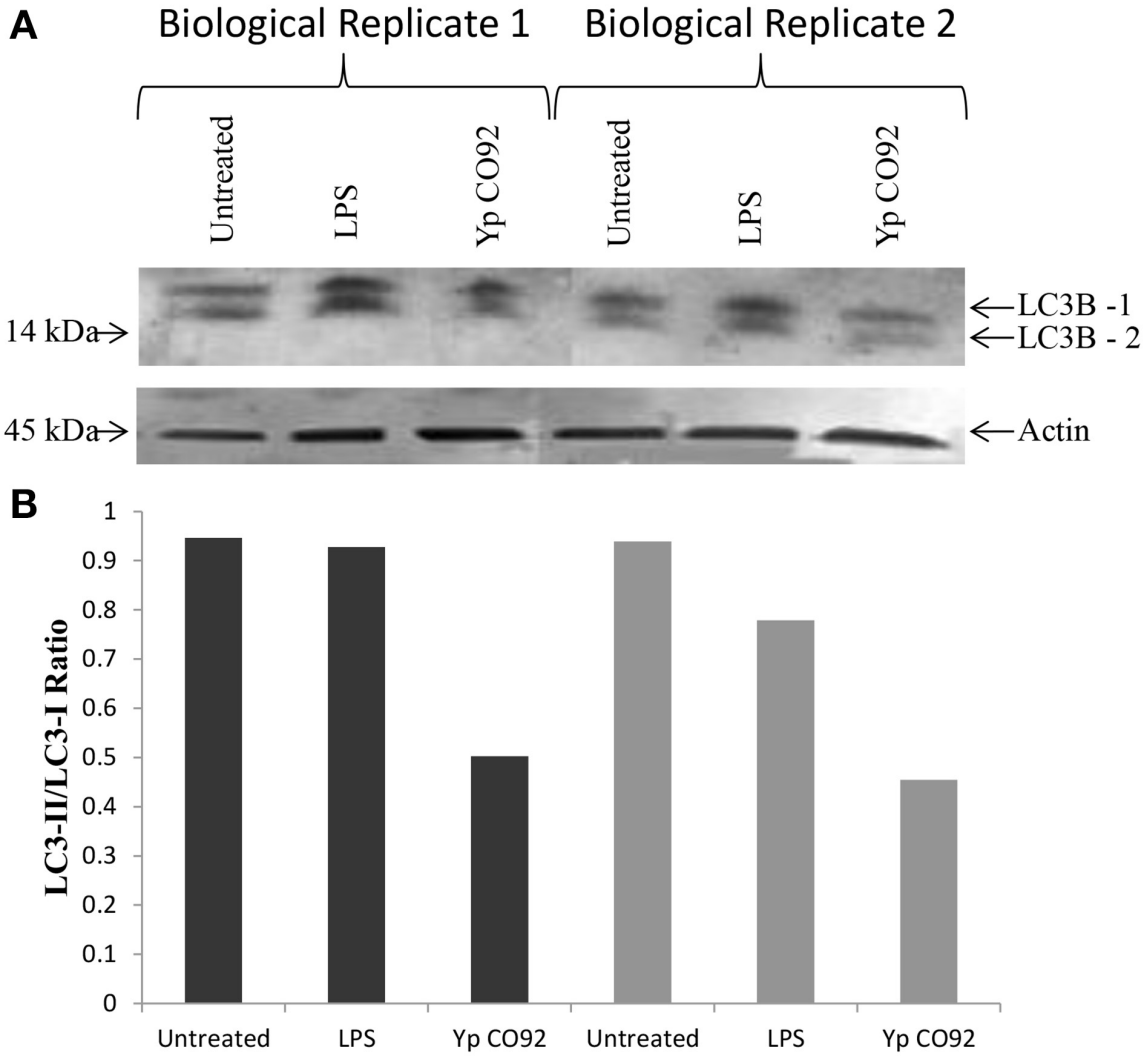
**Decreased conversion of LC3B-I to LC3B-II during Yp infection. (A)** LC3-B Western blot analysis of total cell extracts obtained from HBE cells at 8 h post infection with CO92, or post treatment with LPS, compared to the uninfected and untreated control. The results for two sets of biological replicates are shown. Actin levels were also probed for all samples to serve as loading control and allow normalization of the signals. **(B)** Bar graph showing LC3II/LC3I ratios for all samples based on the densitometry analysis for the LC3I and LC3II protein bands.

**Figure 5:**
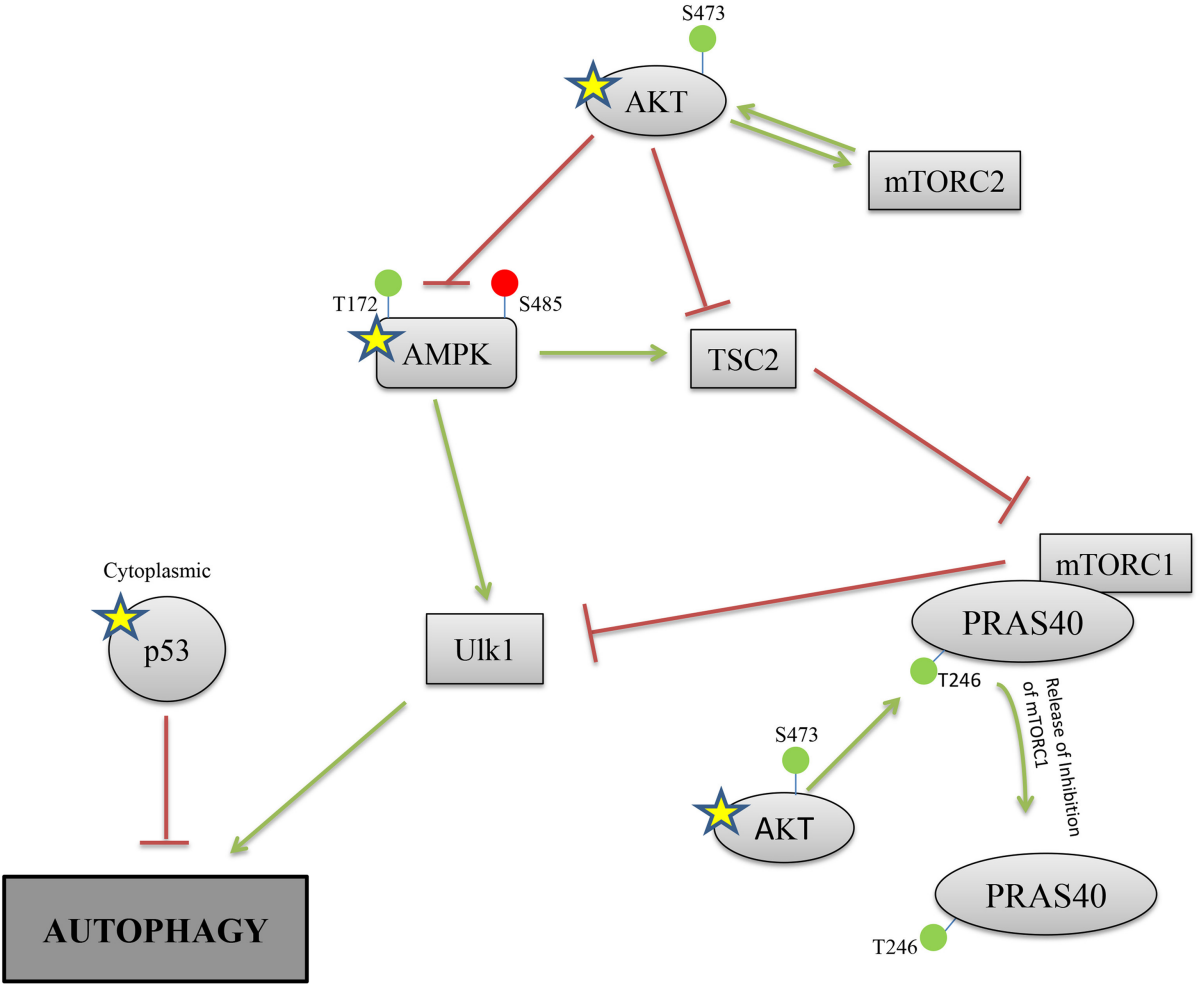
**Proposed model of negative regulation of autophagy during Yp infection**. Pathway map for regulation of the autophagy process highlights proteins that were identified by the RPMA analysis (Yellow star). Red lines depict inhibitor signals and green lines depict activation. The RPMA results demonstrate activation of AKT and p53, and inhibition of AMPKα-1, during Yp infection. In addition, immunofluorescence data (Figure 3) shows a dramatic increase in cytoplasmic localization of p53 protein during Yp infection, and also the LC3-I to LC3-II conversion is significantly reduced (Figure 4). These findings together suggest coordinate negative regulation of autophagy in human bronchial epithelial cells following infection with Yp.

Several identified hits are proteins that are known to regulate the autophagy process; pAKT (S473 and T308), AMPKalpha-1 (S485), and p53 (S15). Based on our RPMA analysis, the AKT kinase is activated during infection with Yp through phosphorylation of its S473 residue whereas AMPK activity is lowered through phosphorylation of its S485 residue, which is known to lead to AMPK inactivation (Ning et al., 2011). Because activated AKT is an inhibitor of autophagy whereas activated AMPK is an inducer, these results suggest that autophagy is down-regulated during the first hours post Yp infection. Our results also show that the levels of p53, another known regulator of autophagy, are significantly increased during Yp infection. Because studies have shown that cytoplasmic localization of p53 down-regulates autophagy (Liang and Clarke, 2001; Tasdemir et al., 2008b), we analyzed the nuclear versus cytoplasmic localization of p53 post Yp infection. Confocal microscopy analysis showed that while prior to infection p53 shows nuclear localization, subsequent to Yp infection activated p53 (S15) shows significantly increased localization in the cytoplasm compared to uninfected controls (Figure 3A). Specifically, 91% of infected cells examined showed a clear presence of p53 in the cytoplasm, compared to only 15% for the uninfected cells (Figure 3B), consistent with a negative regulation of autophagy. We also analyzed the LC3 conversion from LC3-I to LC3-II during Yp infection. Conversion from LC3-I to LC3-II occurs through conjugation of phosphatidylethanolamine (PE) to LC3-I and is a marker for induction of autophagy (Tanida et al., 2008). This conjugation leads to the recruitment of LC3-II to the phagosomal membrane, and as autophagy is induced the ratio of LC3-II to LC3-I increases. Our data shows that after infection with Yp the ratio of LC3-II/LC3-I decreases approximately 2-fold in comparison to the uninfected/untreated condition and also the LPS treatment condition (Figure 4), indicating an inhibition of autophagy. Together, the LC3 and p53 results are consistent with a model of negative regulation of autophagy following Yp infection and suggest a coordinated down-regulation through activation of AKT, inhibition of AMPK, and cytoplasmic localization of p53 (Figure 5).

## Discussion

We took advantage of the high throughput assay format and high sensitivity and reliability of the RPMA platform to quantitatively profile the host response to *Y. pestis* infection, particularly the signaling pathways that become modulated. Because the RPMA platform is amenable to simultaneous analysis of numerous pathways, it affords the possibility of measuring the overall signaling network changes that occur in the cell. For measuring changes in phosphorylation states or total protein levels, we probed with a total of 111 antibodies that span multiple functional categories relevant to infection processes, including apoptosis and survival, autophagy, cell cycle regulation and cellular growth, cytoskeleton modulation and cellular migration, immune response, and chromatin modulation. One of the cell types that we analyzed was the human bronchial epithelial cell line 16HBE14o-, which has been demonstrated to serve as a model system of the airways (Forbes et al., 2003). We used both a fully virulent strain of Yp, CO92, and a derivative avirulent strain for the infections and also profiled changes that can be elicited in a TTSS-independent manner.

### Implication of autophagy, cell cycle and growth, and survival-related pathways

Compared to the control condition (untreated and uninfected cells), a total of 25 proteins in 16HBE14o- cells showed significant fold change in response to the treatment conditions. Survival-related pathways, and pathways involved in regulation of autophagy or cell cycle and growth, are strongly highlighted by the RPMA results. Interestingly, the functional consequences of the RPMA changes observed for category 1 hits, which represent responses observed only for infection with the virulent Yp strain, point to an overall activation of survival functions. Thus, activated AKT (S743), phosphorylated Ras-GRF1 (S916) (activator of Ras), and phosphorylated BAD (S155), are all involved in cell survival pathways (Datta et al., 1997; Brunet et al., 1999; Downward, 2004; Jeon et al., 2012). In addition, the increased levels of active CHK-1 (S345) (DNA damage cell cycle checkpoint) also points toward activation of survival pathways (Liou et al., 2014; Sarmento et al., 2014; Wang et al., 2014).

Category 2 proteins also strongly point to regulation of survival-related pathways, although unlike category 1 proteins the observed RPMA changes highlight more the activation of cell death pathways. In addition, inhibition of cell growth and proliferation is suggested by some of the observed changes. Thus, the specific modulations observed for 12 of the 15 proteins in this category suggest activation of apoptotic functions (Evan et al., 1992; Packham and Cleveland, 1995; Verheij et al., 1996; Hakem et al., 1998; Janicke, 1998; Boulares et al., 1999; Porter and Jänicke, 1999; Ruiter et al., 1999; Allsopp et al., 2000; Slee et al., 2001; Marsden et al., 2002; Amaral et al., 2010). From this group of proteins, ERK (T202/Y204), Gab1 (Y627), SHP2 (Y580), and SHIP1 (Y1020) have also been implicated for cell growth (Hakak et al., 2000; Araki et al., 2003; Ivins Zito et al., 2004; Mattoon et al., 2004; Gloire et al., 2006; Lu and Xu, 2006; Mood et al., 2006; Balmanno and Cook, 2009; Eulenfeld and Schaper, 2009). Since all of these 4 proteins show down-regulation in response to Yp infection, these results also provide evidence for a potential down-regulation of cell growth as part of the host response to Yp. Since both categories 1 and 2 profile significant host responses under functional TTSS conditions, the observed changes may be related to the functions of the Yop effector proteins although clearly further mechanistic studies are needed to address this question. In addition, it remains to be investigated which of the host pathway changes that we have observed by our RPMA analysis reflect Yp manipulation of the host machinery and which are reflections of direct host response.

Treatments with heat-killed CO92 strain and with LPS allowed profiling host responses that can occur independently of the TTSS machinery. Similar to the findings for category 1 and category 2, changes observed in response to treatment with heat killed Yp or LPS suggest activation of survival-related pathways. For example, BAD (Bcl-2 Associated Death) is a protein that induces apoptosis by blocking BAX from binding to Bcl-2 or Bcl-XL, and allows cytochrome C release from mitochondria to activate the intrinsic death pathway. Phosphorylation of the BAD protein at Ser 136, Ser 112, or Ser 155, which are observed in our RPMA analysis, has been shown to inactivate BAD, and therefore promote cell survival (Datta et al., 1997; Peso, 1997; Brunet et al., 1999). Similarly, the observed increased levels of activated CREB (S133) is known to result in increased cell proliferation and survival (Shaywitz and Greenberg, 1999; Mayr and Montminy, 2001). GSK-3α, a serine-threonine kinase, which influences multiple downstream pathways ranging from cell survival/apoptosis to growth and immune response (Ohteki, 2000; Downward, 2004; Tseng et al., 2006; Kotliarova et al., 2008), can also be modulated through TTSS-independent mechanisms. Thus, in response to heat-killed Yp treatment, GSK-3α shows increased phosphorylation at S21 residue during early post infection times (1 and 4 h post infection), which is known to inactivate GSK-3α (Kotliarova et al., 2008). Consistent with our interpretations for the observed changes for the BAD and CREB proteins, the inactivation of GSK-3α may also be pointing to promotion of survival.

### Model for negative regulation of the autophagy pathway

Several of our identified hits are known to modulate the autophagy process. Autophagy is a cellular mechanism that can be utilized by the host to eliminate invading pathogens but it can also be manipulated by the pathogen to survive and replicate (Owen et al., 2014). *Yersinia pestis* has previously been shown to reside in autophagosomes of mouse macrophages in order to evade host immune responses (Pujol et al., 2009). In our study, we observe that multiple proteins that are known to modulate the autophagy pathway are altered in infected cells; pAKT (S743 and T380), AMPKα-1 (S485), and p53 (S15) all showed increased levels at 8 h post infection. In active form, AMPK is a known inducer of autophagy through its activation of ULK1 as well as its role in blocking inhibition of autophagy by mTOR (Xu et al., 2014). Activated AKT (phosphorylated at S473) can phosphorylate AMPK at its S485 residue, leading to AMPK inactivation and therefore inhibition of autophagy (Ning et al., 2011). In addition, AKT directly blocks activation of TSC2 (an inhibitor of mTOR and inducer of autophagy) to provide further regulation of the autophagy process (Inoki et al., 2002; Cai et al., 2006). The p53 protein is another RPMA hit that has also been shown to play a role in autophagy. Cytoplasmic p53 has been shown to repress autophagy (O'Brate and Giannakakou, 2003; Mariño et al., 2014) whereas nuclear p53 induces autophagy through transactivation of genes that suppress mTOR (Tasdemir et al., 2008a). Together, our RPMA results (AKT and p53 activation, and AMPKα-1 inactivation), along with our demonstration of the cytoplasmic localization of p53 and reduced LC3-I to LC3-II conversion during Yp infection, provide evidence of negative regulation of autophagy as part of the host response to Yp (Figure 5). It is of significance that the RPMA changes are mostly observed in response to the virulent Yp strain, and therefore negative regulation of autophagy may represent an important mechanism in determination or regulation of virulence. Further mechanistic studies are required to strengthen our autophagy model and to investigate whether it plays a role in regulation of Yp virulence, in particular given the role of autophagy in bacterially infected cells (Yuk et al., 2012) and the reported findings that intracellular Yp bacteria residing in autophagosomes can avoid xenophagy (Pujol et al., 2009).

### Discovery of novel hits and validation of previous studies in the context of Yp infection

Our RPMA analysis led to the identification of 12 novel hits with respect to Yp infection. Consistent with the reported importance of autophagy and apoptosis/survival pathways during Yp infection (Pujol et al., 2009; Ke et al., 2013; Peters et al., 2013; Caulfield et al., 2014), many of the novel hits implicate these pathways. Furthermore, the cell cycle and growth control pathways are implicated through modulation of proteins such as Chk-1 (S345), c-Myc, SHIP1 (Y1020), or SHP2 (Y580) given the reported functions of these proteins (Schmidt, 1999; Liu et al., 2000; Gloire et al., 2006; Yang et al., 2006; Niida et al., 2007; Tsang et al., 2012). A level of validation of our RPMA results comes from identification of several hits that have been previously reported to be relevant (Table 3). For instance, the RPMA hits Gab1, Gab2, and Lck have been shown to be targeted by YopH in order to regulate the host immune response (De la Puerta et al., 2009). Or, extending previous findings for infection with *Y. pseudotuberculosis* (Uliczka et al., 2009), our RPMA results show that the AKT kinase is also activated during infection with Yp. However, the majority of the previous reports related to our RPMA hits have been either in relation to infection with other Yersinia species or within the context of using recombinant proteins and transfections with specific Yp components (Table 3). Therefore, beyond providing a level of validation, our identification of several reported changes also places a number of the previous findings within the more relevant context of whole infection with Yp.

With continuing significant concern over the rising level of antibiotic resistance for many bacterial infections, focusing on development of host-based therapies founded on an understanding of the host response mechanisms provides a viable alternative for devising effective countermeasures. In addition, a better understanding of the host response networks will allow advances in development of much needed vaccines and early diagnostic measures. The type of high throughput host protein analysis that is afforded by RPMA and has been presented here provides a unique opportunity to measure protein network changes that occur across the host cell in response to infection. The RPMA results presented here should provide a strong foundation for further mechanistic studies of Yp infection, and help pave the way for devising novel effective countermeasures for treatment and prevention of plague.

### Conflict of interest statement

The Associate Editor Fatah Kashanchi declares that, despite being affiliated to the same institution as authors Farhang Alem, Kuan Yao, Valarie Calvert, Emanuel F. Petricoin, Liana Kramer and Ramin M. Hakami, the review process was handled objectively and no conflict of interest exists. The authors declare that the research was conducted in the absence of any commercial or financial relationships that could be construed as a potential conflict of interest.
