# Supplementary material for: Host response during Yersinia pestis infection of human bronchial epithelial cells involves negative regulation of autophagy and suggests a modulation of survival-related and cellular growth pathways
Source: Front Microbiol. 2015 Feb 13;6:50. doi: 10.3389/fmicb.2015.00050 (PMC4327736; doi:10.3389/fmicb.2015.00050)
Supplement: Supplementary file 1 [file DataSheet1.DOCX]

**Supplementary Table 1. List of RPMA antibodies and the cellular pathways of their target proteins.** All **t**he RPMA antibodies that were evaluated are listed and separated based on the specific pathways in which their target proteins are involved. For each antibody, the name of the vendor company, vendor catalog number, and protein band sizes in kilodalton (kDa), are indicated. Antibody dilutions for Western blot validation are also listed.

| **Cell Cycle Regulation and Growth** | **Catalog #** | **Company** | **MW (kDa)** | **Dilutions** |
| --- | --- | --- | --- | --- |
| Akt | 9272 | Cell Signaling | 60 | 1:1000 |
| Akt (S473) | 9271 | Cell Signaling | 60 | 1:1000 |
| Akt (T308) | 9275 | Cell Signaling | 60 | 1:1000 |
| AMPKα1 (S485) | 4184 | Cell Signaling | 62 | 1:1000 |
| ATF-2 (T71) | 9221 | Cell Signaling | 70 | 1:1000 |
| ATF-2 (T69/T71) | 9225 | Cell Signaling | 70 | 1:500 |
| Aurora A (T288)/B (T232)/C (T198) | 2914 | Cell Signaling | 35, 40, 48 | 1:500 |
| c-Abl (T735) | 2864 | Cell Signaling | 120 | 1:1000 |
| c-Abl (Y245) | 2861 | Cell Signaling | 135 | 1:1000 |
| CDK2 (78B2) | 2546 | Cell Signaling | 33 | 1:1000 |
| Chk1 (S345) | 2341 | Cell Signaling | 56 | 1:1000 |
| cMyc | 9402 | Cell Signaling | 57-70 | 1:500 |
| Cyclin D1 (T286) | 2921 | Cell Signaling | 36 | 1:1000 |
| CREB | 9192 | Cell Signaling | 43 | 1:1000 |
| CREB (S133) | 9191 | Cell Signaling | 43 | 1:1000 |
| EGFR (Y1148) | 4404 | Cell Signaling | 175 | 1:500 |
| eIF4G | 2498 | Cell Signaling | 220 | 1:1000 |
| Elk-1 (S383) | 9828 | Cell Signaling | 62 | 1:1000 |
| eNOS (S1177) | 9571 | Cell Signaling | 140 | 1:1000 |
| ERK 1/2 | 9102 | Cell Signaling | 42, 44 | 1:1000 |
| ERK 1/2 (T202/Y204) | 9101 | Cell Signaling | 42, 44 | 1:1000 |
| FAK (Y397) (18) | 611806 | BD | 125 | 1:1000 |
| FAK (Y576/577) | 3281 | Cell Signaling | 125 | 1:1000 |
| GAB1 (Y627) | 3231 | Cell Signaling | 110 | 1:1000 |
| GSK-3α (S21) (46H12) | 9337 | Cell Signaling | 51 | 1:2000 |
| GSK-3α (Y279)/beta (Y216) | 44-604 | Bio Source | 47, 51 | 1:1000 |
| GSK-3α/β (S21/9) | 9331 | Cell Signaling | 46, 51 | 1:1000 |
| GSK-3β (S9) | 9336 | Cell Signaling | 46 | 1:1000 |
| GSK3-β | 9332 | Cell Signaling | 46 | 1:1000 |
| MEK1/2 | 9122 | Cell Signaling | 45 | 1:1000 |
| MEK1/2 (S217/221) | 9121 | Cell Signaling | 45 | 1:1000 |
| mTOR | 2972 | Cell Signaling | 289 | 1:1000 |
| p53 | 9282 | Cell Signal | 53 | 1:1000 |
| p53 (S15) | 9284 | Cell Signaling | 53 | 1:1000 |
| p70 S6 Kinase (S371) | 9208 | Cell Signaling | 70, 85 | 1:1000 |
| p90RSK (S380) | 9341 | Cell Signaling | 90 | 1:1000 |
| PI3-Kinase | 610045 | BD | 85 | 1:2500 |
| PKC (pan) (betaII S660) | 9371 | Cell Signaling | 78, 80, 82, 85 | 1:1000 |
| PKC delta (T505) | 9374 | Cell Signaling | 78 | 1:1000 |
| PLK1 (T210) | 558400 | BD | 68 | 1:10,000 |
| Raf (S259) | 9421 | Cell Signaling | 74 | 1:500 |
| Ras-GRF1 (S916) | 3321 | Cell Signaling | 155 | 1:1000 |
| Rb (S780) | 8180 | Cell Signaling | 110 | 1:1000 |
| Ribosomal protein L13a | 2765 | Cell Signaling | 110 | 1:1000 |
| SAPK/JNK | 9252 | Cell Signaling | 46, 54 | 1:1000 |
| SAPK/JNK (T183/Y185) | 9251 | Cell Signaling | 46, 54 | 1:1000 |
| SHIP1 (Y1020) | 3941 | Cell Signaling | 145 | 1:1000 |
| SHP2 (Y580) | 44-558 | Biosource | 70 | 1:500 |
| Smad1/5 (S463/S465) /Smad9 (S465/S467) | 9511 | Cell Signaling | 60 | 1:500 |
| Src Family (Y416) | 2101 | Cell Signaling | 60 | 1:1000 |
| Ubiquitin (P4D1) | 3936 | Cell Signaling | Many | 1:500 |
| **Cell Survival and Apoptosis** | **Catalog #** | **Company** | **MW (kDa)** | **Dilutions** |
| Akt | 9272 | Cell Signaling | 60 | 1:1000 |
| Akt (S473) | 9271 | Cell Signaling | 60 | 1:1000 |
| Akt (T308) | 9275 | Cell Signaling | 60 | 1:1000 |
| AMPKα1 (S485) | 4184 | Cell Signaling | 62 | 1:1000 |
| ASK1 (S83) | 3761 | Cell Signaling | 155 | 1:1000 |
| ATF-2 (T71) | 9221 | Cell Signaling | 70 | 1:1000 |
| ATF-2 (T69/T71) | 9225 | Cell Signaling | 70 | 1:500 |
| Bad (S112) | 9291 | Cell Signaling | 23 | 1:1000 |
| Bad (S136) | 9295 | Cell Signaling | 23 | 1:500 |
| Bad (S155) | 9297 | Cell Signaling | 23 | 1:1000 |
| Bcl-2 (S70) (5H2) | 2827 | Cell Signaling | 28 | 1:1000 |
| Bcl-2 (T56) | 2875 | Cell Signaling | 28 | 1:1000 |
| c-Abl (T735) | 2864 | Cell Signaling | 120 | 1:1000 |
| c-Abl (Y245) | 2861 | Cell Signaling | 135 | 1:1000 |
| Caspase-3, cleaved (D175) | 9661 | Cell Signaling | 17, 19 | 1:1000 |
| Caspase-3, cleaved (D175) (5A1) | 9664 | Cell Signaling | 17, 19 | 1:1000 |
| Caspase-6, cleaved (D162) | 9761 | Cell Signaling | 18 | 1:1000 |
| Caspase-7, cleaved (D198) | 9491 | Cell Signaling | 20 | 1:1000 |
| Caspase-9, cleaved (D315) | 9505 | Cell Signaling | 35 | 1:1000 |
| Chk1 (S345) | 2341 | Cell Signaling | 56 | 1:1000 |
| cMyc | 9402 | Cell Signaling | 57-70 | 1:500 |
| CREB | 9192 | Cell Signaling | 43 | 1:1000 |
| CREB (S133) | 9191 | Cell Signaling | 43 | 1:1000 |
| ERK 1/2 | 9102 | Cell Signaling | 42, 44 | 1:1000 |
| Erb-B2 (Y877) | 2245 | Cell Signaling | 185 | 1:1000 |
| FADD (S194) | 2781 | Cell Signaling | 28 | 1:1000 |
| FAK (Y397) (18) | 611806 | BD | 125 | 1:1000 |
| FAK (Y576/577) | 3281 | Cell Signaling | 125 | 1:1000 |
| GSK-3α (S21) (46H12) | 9337 | Cell Signaling | 51 | 1:2000 |
| GSK-3α (Y279)/beta (Y216) | 44-604 | Bio Source | 47, 51 | 1:1000 |
| GSK-3α/β (S21/9) | 9331 | Cell Signaling | 46, 51 | 1:1000 |
| GSK-3β (S9) | 9336 | Cell Signaling | 46 | 1:1000 |
| GSK3-β | 9332 | Cell Signaling | 46 | 1:1000 |
| MDM2 (S166) | 3521 | Cell Signaling | 90 | 1:1000 |
| MSK1 (S360) | 9594 | Cell Signaling | 90 | 1:1000 |
| mTOR (S2448) | 2971 | Cell Signaling | 289 | 1:1000 |
| p38 MAP Kinase | 9212 | Cell Signaling | 40 | 1:1000 |
| p38 MAP Kinase (T180/Y182) | 9211 | Cell Signaling | 40 | 1:1000 |
| p53 | 9282 | Cell Signal | 53 | 1:1000 |
| p53 (S15) | 9284 | Cell Signaling | 53 | 1:1000 |
| PARP, cleaved (D214) | 9541 | Cell Signaling | 89 | 1:1000 |
| PI3-Kinase | 610045 | BD | 85 | 1:2500 |
| Protein phosphatase 1 β | Ab53315 | Abcam | 37 | 1:10,000 |
| PTEN (S380) | 9551 | Cell Signaling | 54 | 1:1000 |
| PTEN | 9188 | Cell Signaling | 54 | 1:1000 |
| pY100 | 9411 | Cell Signaling | 100 | 1:1000 |
| Rb (S780) | 8180 | Cell Signaling | 110 | 1:1000 |
| SAPK/JNK | 9252 | Cell Signaling | 46, 54 | 1:1000 |
| SAPK/JNK (T183/Y185) | 9251 | Cell Signaling | 46, 54 | 1:1000 |
| SHIP1 (Y1020) | 3941 | Cell Signaling | 145 | 1:1000 |
| SHP2 (Y580) | 44-558 | Biosource | 70 | 1:500 |
| Ubiquitin (P4D1) | 3936 | Cell Signaling | Many | 1:500 |
| **Immune Response** | **Catalog #** | **Company** | **MW (kDa)** | **Dilutions** |
| GSK-3α (S21) (46H12) | 9337 | Cell Signaling | 51 | 1:2000 |
| GSK-3α (Y279)/beta (Y216) | 44-604 | Bio Source | 47, 51 | 1:1000 |
| GSK-3α/β (S21/9) | 9331 | Cell Signaling | 46, 51 | 1:1000 |
| GSK-3β (S9) | 9336 | Cell Signaling | 46 | 1:1000 |
| GSK3-β | 9332 | Cell Signaling | 46 | 1:1000 |
| IL-6 | 5143-100 | Bio Vision | 21-28 | 1:1000 |
| iNOS | 2977 | Cell Signaling | 130 | 1:500 |
| IκB-α | 551818 | BD | 42 | 1:500 |
| IκB-α (S32/36) (5A5) | 9246 | Cell Signaling | 40 | 1:2000 |
| JAK1 (Y1022/1023) | 3331 | Cell Signaling | 130 | 1:1000 |
| Lck | 2752 | Cell Signaling | 56 | 1:1000 |
| Lck (Y505) | 44-850 | Biosource | 56 | 1:1000 |
| NFkB | 3034 | Cell Signaling | 75 | 1:1000 |
| NF-κB p65 (S536) | 3032 | Cell Signaling | 65 | 1:1000 |
| PKC (pan) (betaII S660) | 9371 | Cell Signaling | 78, 80, 82, 85 | 1:1000 |
| PKC θ (T538) | 9377 | Cell Signaling | 79 | 1:1000 |
| PKCα | 3550 | Upstate | 82 | 1:1000 |
| Stat1 | 9172 | Cell Signaling | 84, 91 | 1:1000 |
| Stat1 (Y701) | 9171 | Cell Signaling | 84, 91 | 1:1000 |
| Stat1 (Y701) | 07-307 | Upstate | 92 | 1:1000 |
| Stat3 (S727) | 9134 | Cell Signaling | 79, 86 | 1:1000 |
| Stat3 (Y705) (9E12) | 05-485 | Upstate | 92 | 1:10,000 |
| **Cytoskeletal Rearrangement, Cell Migration, and Vesicle Trafficking** | **Catalog #** | **Company** | **MW (kDa)** | **Dilutions** |
| Actin, β | 4967 | Cell Signaling | 45 | 1:1000 |
| AMPKa1(S485) | 4184 | Cell Signaling | 62 | 1:1000 |
| Catenin-Beta (S33/37/T41) | 9561 | Cell Signaling | 85 | 1:1000 |
| Cofilin (S3) (77G2) | 3313 | Cell Signaling | 19 | 1:1000 |
| FAK | 556368 | BD | 116 | 1:1000 |
| FAK (Y397) (18) | 611806 | BD | 125 | 1:1000 |
| FAK (Y576/577) | 3281 | Cell Signaling | 125 | 1:1000 |
| GSK-3α (S21) (46H12) | 9337 | Cell Signaling | 51 | 1:2000 |
| GSK-3α (Y279)/beta (Y216) | 44-604 | Bio Source | 47, 51 | 1:1000 |
| GSK-3α/β (S21/9) | 9331 | Cell Signaling | 46, 51 | 1:1000 |
| GSK-3β (S9) | 9336 | Cell Signaling | 46 | 1:1000 |
| GSK3-β | 9332 | Cell Signaling | 46 | 1:1000 |
| HSP70 (C92F3A-5) | SPA-810 | Stressgen | 70 | 1:2000 |
| HSP90a (T5/7) | 3488 | Cell Signaling | 90 | 1:500 |
| JAK1 (Y1022/1023) | 3331 | Cell Signaling | 130 | 1:1000 |
| LIMK1 (T508)/LIMK2 (T505) | 3841 | Cell Signaling | 72 | 1:1000 |
| PLC-γ-1 | 2822 | Cell Signaling | 155 | 1:1000 |
| PLC-γ-1 (Y783) | 2821 | Cell Signaling | 155 | 1:1000 |
| Vav3 (Y173) | 44-488 | Biosource | 95 | 1:500 |
| **Chromatin Modulation** | **Catalog #** | **Company** | **MW (kDa)** | **Dilutions** |
| Histone H3, acetyl- (Lys14) | 07-353 | Millipore | 17 | 1:1000 |
| Histone H3, Di-Methyl (Lys9) | 9753 | Cell Signaling | 15 | 1:500 |
| Histone H3, Di-Methyl (Lys27) | 9755 | Cell Signaling | 15 | 1:500 |
| Histone H3, Pan-Methyl (Lys9) | 4069 | Cell Signaling | 15 | 1:500 |
| Histone H3, tri-methyl (Lys27) | 07-449 | Millipore | 17 | 1:5000 |
| Histone H3 | Ab1791 | Abcam | 17 | 1:500 |
| Histone H3 (S10) | 06-570 | Upstate | 17 | 1:1000 |
| PCAF (C14G9) | 3378 | Cell Signaling | 93 | 1:500 |
| SUMO-1 | 4930 | Cell Signaling | Many | 1:500 |
| SUMO 2-3 | 4971 | Cell Signaling | Many | 1:500 |
| **Autophagy** | **Catalog #** | **Company** | **MW (kDa)** | **Dilutions** |
| p53 (S15) | 9284 | Cell Signaling | 53 | 1:1000 |
| p53 | 9282 | Cell Signaling | 53 | 1:1000 |
| Akt (S473) | 9271 | Cell Signaling | 60 | 1:1000 |
| Akt (T308) | 9275 | Cell Signaling | 60 | 1:1000 |
| Akt | 9272 | Cell Signaling | 60 | 1:1000 |
| c-Abl (Y245) | 2861 | Cell Signaling | 135 | 1:1000 |
| eNOS (S1177) | 9571 | Cell Signaling | 140 | 1:1000 |
| p90RSK (S380) | 9341 | Cell Signaling | 90 | 1:1000 |
| Atg5 | 2630 | Cell Signaling | 55 | 1:500 |
| LC3B | 2775 | Cell Signaling | 14,16 | 1:500 |
| AMPKα1 (S485) | 4184 | Cell Signaling | 62 | 1:1000 |
| mTOR | 2972 | Cell Signaling | 289 | 1:1000 |
